# USP39 at the crossroads of cancer immunity: regulating immune evasion and immunotherapy response through RNA splicing and ubiquitin signaling

**DOI:** 10.3389/fimmu.2025.1665775

**Published:** 2025-09-08

**Authors:** Feilong Zhou, Xinhao Li, Yanmei Sun, Yizhu Wang, Kaiyi Niu, Xin Gao, Jiaqi Zhang, Tianyi Chen, Yunxin Li, Weijie Zhao, Binyue Mao, Qiyang Xu, Yanlong Shi, Zhenyu He

**Affiliations:** ^1^ General Surgery Department, The Second Affiliated Hospital of Nanjing Medical University, Nanjing, China; ^2^ Department of Dermatology, The First Affiliated Hospital of Xi’an Jiaotong University, Xi’an, China; ^3^ General Surgery Department, Fuyang Fifth People’s Hospital, Fuyang, China; ^4^ General Surgery Department, Fuyang Hospital of Anhui Medical University, Fuyang, China

**Keywords:** USP39, deubiquitylation, structure, DNA damage repair, RNA splicing, regulation, cancers, immunotherapy

## Abstract

Deubiquitinating enzymes (DUBs) are responsible for the removal of ubiquitin from substrates, thereby antagonizing ubiquitination and regulating a multitude of biological pathways including cell cycle progression, signal transduction, and transcriptional regulation. Ubiquitin Specific Protease-39 (USP39), a pivotal member of the ubiquitin-specific protease family, is intricately linked to innumerable pathophysiological processes. In this review, we first provide an overview of the specific structural domains and biological functions of USP39, with a particular focus on its role in DNA damage repair and RNA splicing processes. Then, we delineate the function of USP39 in maintaining epithelial morphology, resistance to viral infection, vascular remodeling, and pathological states. Moreover, we particularly focus on the aberrant expression of USP39 in various cancers and its effect on cancer markers, as well as on the regulatory role of USP39 in tumor progression. In conclusion, a comprehensive analysis of the structural domains and functional properties of USP39, a detailed investigation into its interaction mechanisms with diverse substrates, and the accelerated development of related inhibitors will provide a novel theoretical foundation for the treatment of numerous diseases, including tumors. Importantly, targeting USP39 may overcome resistance to checkpoint inhibitors, offering a promising approach to enhance cancer immunotherapy efficacy.

## Graphical Abstract

This review provides a comprehensive overview of USP39’s structure, physiological function, and promise as a biomarker in cancer diagnosis and treatment.

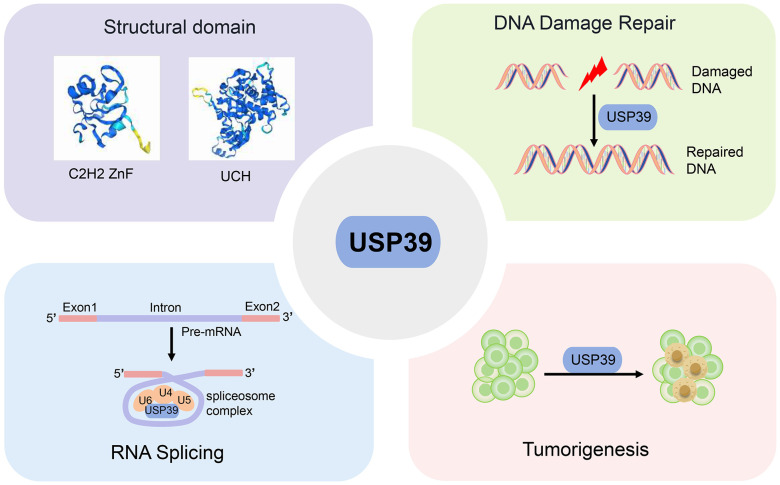

## Introduction

**Figure 1:**
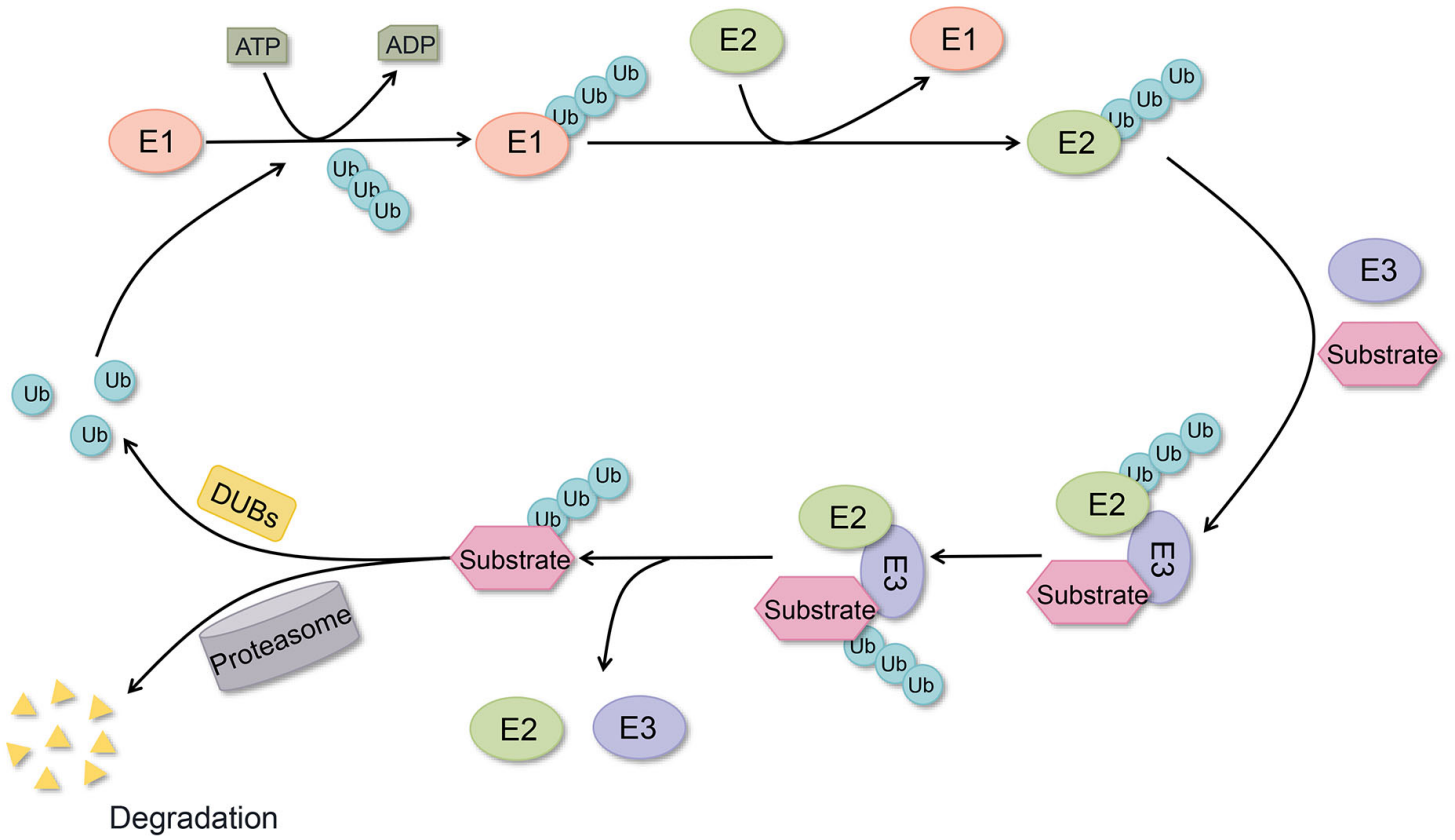
Ubiquitination and deubiquitylation processes. Ubiquitin conjugation to target proteins via E1, E2, and E3 enzymes, with DUBs mediating ubiquitin removal from substrates and ubiquitin recycling.

Proteins serve as the fundamental regulatory units for cellular functions, with ubiquitination representing the second most prevalent post-translational modification (PTM) after phosphorylation (1). A substantial body of evidence indicates that ubiquitination plays a pivotal role in multiple essential intracellular processes, including cell cycle regulation, apoptosis, DNA damage repair, immune regulation, and signaling, among numerous others (2, 3). Ubiquitination modification is a stepwise process carried out by ubiquitin-activating enzyme E1, ubiquitin-conjugating enzyme E2, and ubiquitin-conjugating enzyme E3, which centers on the formation of a stable covalent isopeptide bond between the glycine of ubiquitin (Ub) and the lysine residues of the target protein (4) (**Figure 1**). Ub, a highly conserved small molecule comprising 76 amino acid residues, was initially postulated to be the triggering factor for the degradation of proteins by the 26S proteasome (5). The E3 ubiquitin ligase exerts a crucial influence within the ubiquitinating enzyme complex, as it directly interacts with the substrate and determines the specificity of the ubiquitination reaction (6). Ubiquitination includes several types, and different types of ubiquitination determine different fates of proteins, such as K48 chain-type polyubiquitination labels proteins degraded by the 26S proteasome, while K63 chain-type polyubiquitination is involved in the assembly of signaling complexes and the regulation of signaling pathways (7, 8).

Deubiquitylation represents the inverse process of ubiquitination, which is catalyzed by DUBs (9). At present, about 100 DUBs have been identified in the human genome and can be classified into seven subgroups based on the conservation of their sequences and structural domains. These include the following classes of DUBs: ubiquitin-specific proteases (USPs), ovarian tumor proteases (OTUs), ubiquitin C-terminal hydrolases (UCHs), Machado-Joseph disease protein domain proteases (MJDs), JAB1/MPN+/MOV34 motif proteases (JAMMs), ubiquitin-containing DUB family-interacting motifs (MINDYs), and zinc fingers of proteins with UFM1-specific peptidase structural domains (ZUFSPs) (2). Furthermore, monocyte chemotactic protein-induced proteins (MCPIPs) have been identified as members of the DUBs family, exhibiting novel DUBs structural domains distinct from those observed in the seven aforementioned DUBs (10). In the classification of DUBs, all subgroups are cysteine proteases, except for JAMMs, which belong to the metalloprotease family (11). The removal of substrate protein-tagged Ub or the inhibition of the conversion of Ub from E2 to E3 by DUBs-catalyzed deubiquitylation serves to maintain intracellular homeostasis and plays an important role in tumorigenesis and other disease progression (12). For example, deubiquitylation regulates numerous cellular signaling pathways, including the EGFR, MAPK, and PI3K/AKT/mTOR pathways, as well as transcription factors such as HIF-1, c-MYC, p53, NRF2, and SREBP1. These regulatory processes ultimately influence metabolic reprogramming in cancer cells (13, 14). Moreover, the involvement of DUBs in autophagy, immune checkpoints, DNA methylation, and chromatin remodeling has been substantiated by empirical evidence.

The USPs constitute the largest and most heterogeneous group of DUBs, comprising more than 50 members (15). The defining characteristic of USPs is the presence of a conserved catalytic structural domain, designated the USP structural domain, which encompasses three sub-structural units. These structural domains are analogous to those observed in the human hand, specifically the fingers, thumb, and palm. Collectively, these sub-structural domains endow USPs with proteolytic activity, enabling them to cleave target proteins from Ub molecules (16, 17). In addition to the USP catalytic structural domain, USPs possess other functional structural domains, including the ubiquitin-like (UBL) structural domain, the zinc finger ubiquitin-binding protein (ZnF-UBP) structural domain, and the ubiquitin interaction motif (UIM). These functional structural domains impact substrate recognition, protein-protein interactions, and subcellular localization, thereby enhancing the overall regulation of protein deubiquitylation events by USPs. A substantial body of evidence from previous studies has demonstrated the critical role of USPs in maintaining the dynamic equilibrium of ubiquitination and deubiquitylation. Dysregulation of this process has been linked to a multitude of pathophysiological processes, including inflammation, metabolism, immunity, and cancer drug resistance, among others (18–20). For instance, the upregulation of USP8 in pancreatic cancer has been observed to deubiquitinate PD-L1, which can reduce PD-L1 levels by targeting USP8. This, in turn, has been shown to stimulate cytotoxic T cells and enhance the anti-tumor immune response and the efficacy of PD-L1-targeted immunotherapy (21). USP14 has been demonstrated to lead to downregulation of chemotactic responses to CXCL12 by regulating the deubiquitylation of the chemokine receptor CXCR4. This, in turn, has been shown to regulate the inflammatory response (22). Furthermore, it has been evidenced that the combination of USP7 inhibitors with other immunomodulatory agents or chemotherapy agents can markedly enhance the DNA damage effect and facilitate the overcoming of tumor cell resistance to treatment (23, 24).

Despite the significant advancements in the field of DUBs research over the past few decades, there is still a need for further extensive studies to elucidate their mechanisms of action and clinical applications. At present, a gap exists in the systematic review of USP39. The objective of this review is to provide a comprehensive description of the structural features, functional properties, and regulatory role of USP39 in cellular and physiological processes. Additionally, this review will examine the pathological significance of USP39 in the progression of various cancers and other diseases, with a particular focus on its emerging role in mediating immune evasion and immunotherapy resistance.

## Structure of USP39

**Figure 2:**
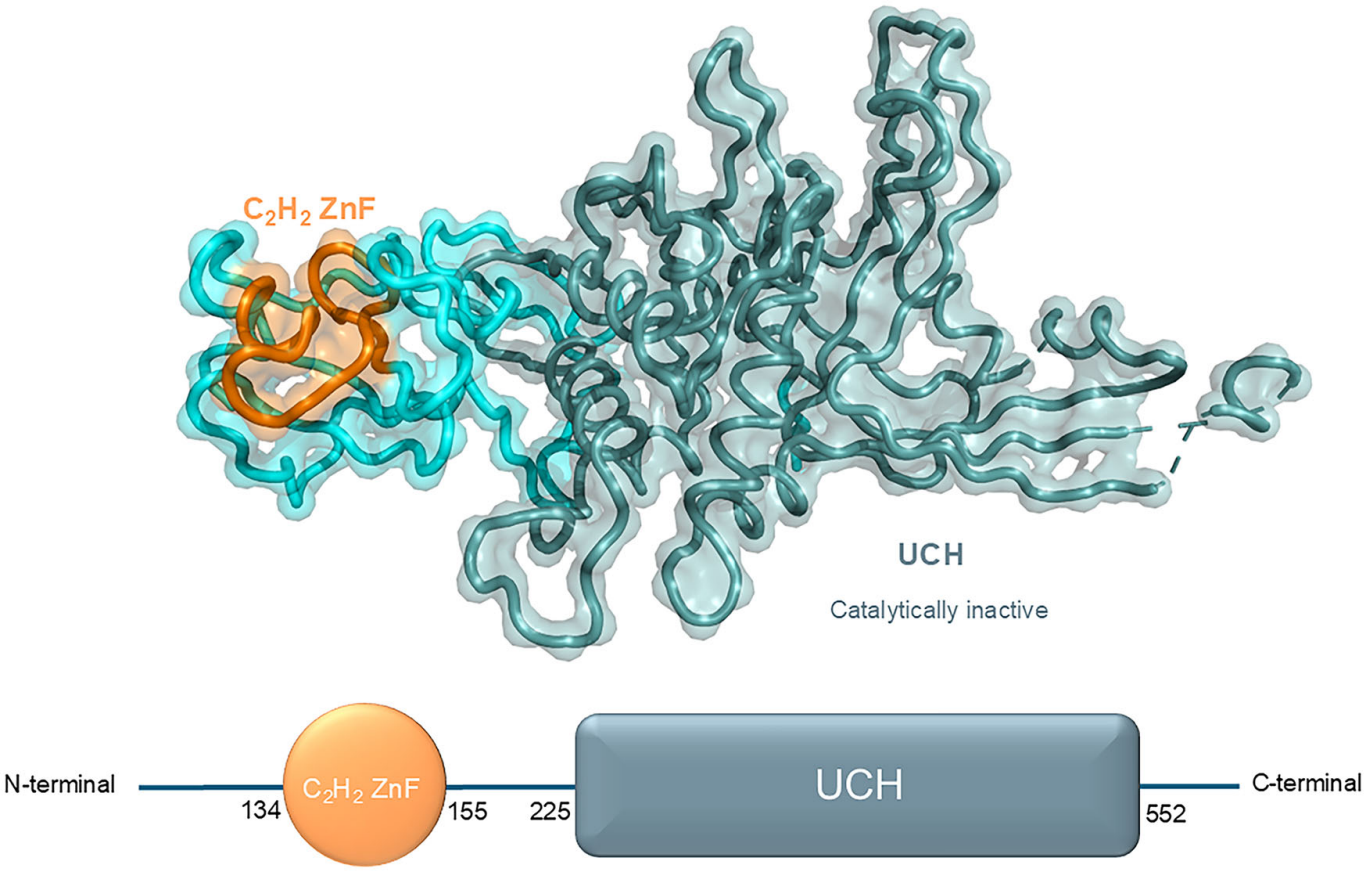
Structural domains and respective crystal structure of USP39 from N-terminal to C-terminal (N-term, 134, 155, 225, 552, C-term).

USP39, also designated U4/U6·U5 tri-snRNP-associated protein 2, is a 565-amino-acid protein that exhibits 65% amino acid identity with yeast SAD1. It comprises two principal structural domains: a zinc finger ubiquitin-binding structural domain (C_2_H_2_ ZnF) and a ubiquitin C-terminal hydrolase (UCH) structural domain (25)(**Figure 2**).

The first class of zinc fingers to be characterized was the C_2_H_2_-type (classical), which is defined by comprising a short β hairpin and an α helix, which together form a β/β/α secondary structure. In this structure, individual zinc atoms are held in place by Cys and His residues in tetrahedral arrays. C_2_H_2_ ZnF can be classified into three groups based on the number and pattern of the fingers: triple C_2_H_2_ (binding a single ligand), multiple neighboring C_2_H_2_s (binding more than one ligand), and separated pairs of C_2_H_2_s (26). The C_2_H_2_ ZnF is the most prevalent DNA-binding motif observed in eukaryotic transcription factors. Additionally, it is present in prokaryotes (27). Transcription factors typically contain multiple ZnFs (each with a conserved β/β/α structure) that are capable of forming multiple contacts along DNA. The C_2_H_2_ ZnF motif can recognize DNA sequences by binding to the major groove of DNA through a short α-helix in the ZnF, which spans 3–4 bases of DNA (28). Furthermore, the C_2_H_2_ ZnF is capable of binding to RNA and protein targets (29). The aforementioned conclusion is supported by the following evidence: Notwithstanding the considerable structural similarity of the C_2_H_2_ ZnF domain of USP39 to other ZnF-UBPs, the ZnF-UBP of USP39 is devoid of the conventional ubiquitin-binding motif. In comparison to the ubiquitin-binding ZnF-UBPs in USP5 and HDAC6, the ZnF-UBP of USP39 exhibits notable distinctions in the conformation of the L2A and β4–5 rings. As a result of the contraction between the L2A and β4–5 loops in USP39, the homologous residue in USP5 responsible for ubiquitin recognition assumes a conformation that impedes the binding of USP39 to ubiquitin (30).

The UCH structural domain represents a distinctive biochemical structural feature of the UCH family, typically present in the protein structures of UCH family members in a conserved structural form. This domain is responsible for mediating deubiquitinating enzyme activity, as evidenced by previous research (31). Notably, the absence of DUB activity was confirmed by *in vitro* analysis of the catalytic structural domain of USP39 toward ubiquitin C-terminal 7-amino-4-methylcoumarin (Ub-AMC), despite the presence of a similar UCH structural domain. In comparison to other active DUB structural domains (e.g., USP4 and USP8), the DUB structural domain of USP39 is devoid of all three catalytic residues. This observation has led to the hypothesis that USP39 is unable to perform ubiquitin cleavage functions *in vivo (* 32). Further analysis of the structure revealed that the UCH structural domain of USP39 forms a characteristic ubiquitin-binding pocket. However, the enzymatic activity of this domain is completely lost due to amino acid substitutions in the active site (30).

Notably, although USP39 contains a DUB domain lacking canonical activity and is classified as inactive, experimental evidence suggests it possesses deubiquitylating activity (33–36). This indicates USP39 may function through non-canonical mechanisms or interactions beyond its catalytic domain, warranting further investigation.

## Biological functions of USP39

Due to its unique structural features, the versatility of USP39 has gradually gained widespread recognition and importance in numerous cellular processes. Specifically, USP39 has been shown to regulate a variety of key cellular processes including DNA damage repair, RNA splicing, epithelial morphogenesis, vascular remodeling, and immune regulation (**Table 1**).

**Table 1:** The potential mechanisms and impacts of USP39 and its substrates in cellular biological processes.

Substrate	Mechanism	Impact	Ref
CHK2	USP39 deubiquitinates CHK2 and enhances CHK2 stability.	Promote cell cycle arrest, apoptosis and DNA repair.	(40)
XRCC4/ LIG4	USP39 acts as a molecular trigger for fluid stratification in a PAR-coupled N46-dependent manner, thereby directly interacting with the XRCC4/LIG4 complex during NHEJ.	Rapidly localize DNA damage in a PAR-dependent manner and regulate DNA damage repair.	(42)
U4/U6·U5 tri-snRNP	USP39 promotes the involvement of U4/U6 in RNA splicing and is involved in U5 tri-snRNP assembly and pre-catalyzed spliceosome formation.	Participate in RNA splicing and ensure correct expression of the coding region of the gene.	(46, 47)
CTLA-4	USP39 increases RNA splicing-mediated CTLA-4 expression and enhances immunosuppression in Treg cells.	Suppress the anti-tumor immunity.	(49)
Hsf1	Deletion of USP39 results in selective 5’ splice site selection for exon 6 in heat shock transcription factor 1 (Hsf1), leading to a decrease in its expression.	Cause lipid accumulation and affect hepatic autophagy.	(51)
Aurora B	USP39 is involved in splicing of Aurora B and other mRNAs.	Maintain the spindle checkpoint and support cytoplasmic division.	(25)
GRHL3	USP39 interacts with GRHL3 and is engaged in the regulation of the subcellular distribution of GRHL3.	Promote epithelial morphogenesis and enhance PCP expression.	(32)
STAT1	USP39 interacts with STAT1 and significantly reduces ubiquitination of the K6-linkage, but not the K48-linkage, of STAT1 and maintains the stability of the STAT1 protein.	Facilitate the type I IFN signaling pathway, increase the expression of type I IFN, and exert significant antiviral effects.	(33, 59)
E protein	USP39 interacts with E proteins, reduces polyubiquitination of E proteins, and protects E proteins from E3 ubiquitin ligase RNF5-mediated degradation.	Promote replication of SARS-CoV-2.	(60)
IκBα	USP39 interacts with IκBα and stabilizes basal levels of IκBα by reducing K48-linked polyubiquitination on IκBα.	Negatively regulate NF-κB-mediated inflammatory responses.	(61)

## DNA damage repair

The family of USPs, as members of the classical deubiquitinating enzyme subfamily, has been confirmed in numerous preceding studies to exert a significant influence on the regulation of genetic information. For instance, USP5 has been shown to possess a vital role in gene maintenance through the regulation of MLH1 stability, a crucial protein involved in DNA mismatch repair (37). In contrast, USP39 is known to be involved in the regulation of chromosomal and genetic information.

The serine/threonine kinase CHK2 (checkpoint kinase 2) is a key mediator of the DNA damage response and tumor suppressor and has been implicated in promoting cell cycle arrest, apoptosis, and DNA repair (38). Previous studies have shown that ubiquitination represents a crucial regulatory mechanism for CHK2 (39). USP39 deubiquitinates and stabilizes CHK2, thereby enhancing CHK2 stability. The downregulation of USP39, achieved through the use of short hairpin RNA (shRNA), has been observed to result in the dysregulation of CHK2. Consequently, this leads to the impairment of DNA damage-induced G2/M checkpoints, a reduction in apoptosis, and an increased resistance of cancer cells to chemotherapeutic agents and radiation therapy (40).

The crosstalk between poly ADP-ribose (PAR), activated PAR polymerase 1 (PARP1) metabolites and the DNA repair machinery has been identified as a decisive regulatory mechanism of the DNA damage response (DDR) (41). USP39 is rapidly localized to DNA damage in a PAR-dependent manner, where it regulates non-homologous end-joining (NHEJ) through a tripartite RG motif located at the N-terminal end of the N-terminus, which contains 46 amino acids. Furthermore, USP39 functions as a molecular trigger for fluid partitioning in a PAR-coupled N46-dependent manner, thereby directly interacting with the XRCC4/LIG4 complex during NHEJ. Concurrently, the USP39-associated spliceosome complex regulates homologous recombination repair in a PAR-independent manner (42). These findings offer insights into the mechanistic basis for USP39’s involvement in controlling DNA repair processes in the DDR.

## RNA splicing

The RNA spliceosome is a complex molecular machine whose primary function is the removal of introns from eukaryotic precursor messenger RNAs (pre-mRNAs) and the ligation of exons encoding for proteins to form mature messenger RNAs (mRNAs). This process is of great importance for eukaryotic gene expression, as it ensures the correct expression of the coding region of the gene. Consequently, RNA spliceosomes frequently play a significant role in various pathophysiological processes (43).

U4/U6·U5 tri-snRNP is the largest pre-assembled spliceosome complex, comprising protein and RNA building blocks that become incorporated into the active site following activation. This includes U6 snRNA, which pairs with 5ss and folds to form the active site. In tri-snRNP, the U6 snRNA pairs with the U4 snRNA, which acts as a chaperone to maintain the pre-catalyzed conformation of U6 (44, 45). The U5 snRNA loop 1 is necessary for the attachment of 5’ exons during branching and the alignment of 5’ exons and 3’ exons at exon junctions (46). USP39 is a component of the spliceosome associated with the U4/U6·U5 tri-snRNP, which is primarily responsible for facilitating the involvement of U4/U6 in RNA splicing. Additionally, USP39 is implicated in the assembly of U5 tri-snRNP and the formation of pre-catalyzed spliceosomes (47). USP39 exerts a considerable influence on B-cell development, a process that is mediated by its capacity to regulate immunoglobulin gene rearrangements and chromatin interactions in a manner that is dependent on the spliceosome (48). Moreover, in tumor-infiltrating regulatory T cells (Tregs), USP39 has been identified to increase RNA splicing-mediated CTLA-4 expression, which in turn enhances the immunosuppressive function of Tregs, thereby suppressing anti-tumor immunity. Conversely, deletion of USP39 in Tregs induces a strong anti-tumor immune response by blocking tumor-associated Tregs activity (49). This suggests targeting USP39 could potentiate checkpoint inhibitors by alleviating CTLA-4-driven immunosuppression.

Alternative splicing (AS) represents a decisive mechanism in the regulation of eukaryotic gene expression, enabling a gene to generate a multitude of mature mRNA splice isoforms through diverse splicing modes, thus enhancing protein diversity (50). USP39 regulates AS in several autophagy-related genes, and deletion of USP39 results in selective 5’ splice site selection in exon 6 of heat shock transcription factor 1 (Hsf1), leading to reduced Hsf1 expression. In contrast, the overexpression of Hsf1 was observed to mitigate the accumulation of lipids that was induced by the deficiency of USP39. The above results indicate that USP39-mediated alternative splicing is necessary for the maintenance of hepatic autophagy and lipid homeostasis (51).

Aurora B is a key protein involved in mitosis, acting as part of the Chromosomal Passenger Complex (CPC). The CPC is responsible for regulating spindle function, spindle checkpoint activity, and cytoplasmic division (52). It has been shown that USP39 acts as a splicing factor for Aurora B and other mRNAs, and is essential for maintaining the spindle checkpoint and supporting successful cytoplasmic division (25).

## Epithelial morphogenesis

The growth and deformation of the epithelial layer are fundamental processes that drive morphogenesis, including the formation of protozoal embryos, the development of ducts, and changes in body shape. The coordination between epithelial cell movement, shape changes, intercalation, division, and death drive these complex morphogenetic processes (53, 54). Granulosa head-like factor (GRHL) plays a dual role in epidermal differentiation and morphogenesis, acting both as a nuclear transcription factor and as a cytoplasmic regulator of planar cell polarity (PCP) components (55, 56). It has been suggested that USP39 is a potential GRHL3-interacting protein involved in regulating the subcellular distribution of GRHL3 and is important for the enhancement of PCP expression in epithelial morphogenesis (32).

## Vascular remodeling

Vascular remodeling is a significant contributor to the failure of angioplasty procedures. The proliferation, migration, and apoptosis of vascular smooth muscle cells (VSMCs) represent the primary processes involved in vascular remodeling. The available evidence suggests that USP39 may play a significant role in the pathogenesis of vascular remodeling. The expression of USP39 was found to be upregulated in both the mouse carotid artery ligation model and the porcine vein graft model. Upregulated USP39 promoted the proliferation and migration of VSMCs, whereas the knockdown of USP39 suppressed the expression of the key proteins of the G1/S-phase transition of the cell cycle, cyclin D1, and CDK4. This indicates that USP39 may represent a potential target for the development of novel therapeutic strategies to treat vascular injury and prevent vein graft failure (57, 58).

## Immunity regulation

In the host antiviral defense mechanism, USP39 interacts with STAT1, resulting in a notable reduction in the ubiquitination of the K6-linkage, but not the K48-linkage, of STAT1. This process maintains the stability of the STAT1 protein and reduces its degradation. STAT1 is a key component that activates the interferon-stimulated response element (ISRE) promoter and induces the expression of interferon-stimulated genes (ISGs) (33, 59). It can therefore be surmised that USP39 may exert significant antiviral effects by promoting the type I interferon (IFN) signaling pathway and increasing the expression of type I IFN. However, it was also found that USP39 interacts with the E protein, a highly conserved envelope protein of SARS-CoV-2, through its N-terminal arginine-rich modality, which reduces polyubiquitination of the E protein and protects it from degradation. The E protein is protected from RNF5-mediated degradation, which enhances its stability and facilitates SARS-CoV-2 replication (60). This indicates that USP39 does not function exclusively as a viral suppressor. The discovery of its multifaceted roles underscores the critical need to evaluate the function of USP39 across various contexts and pathological conditions.

In the inflammatory response, USP39 interacts with IκBα, stabilizing basal levels of IκBα by reducing K48-linked polyubiquitination on IκBα. IκBα is a key protein for inhibiting NF-κB nuclear translocation and NF-κB-mediated signaling. Consequently, USP39 exerts a regulatory effect on NF-κB-mediated inflammatory responses, which is crucial for controlling the activation and recovery of inflammatory responses (61).

## Role of USP39 in cancers

USP39 has a wide range of roles in physiological functions, involving almost all levels of life activities, and is essential for the normal growth and development of organisms. Therefore, dysfunction of USP39 is likely to trigger a wide range of diseases. For example, in the early and presymptomatic stages of Parkinson’s disease, the expression levels of USP39 in the substantia nigra and striatum are significantly decreased (62). Comparatively, USP39 expression is significantly increased in the retinal stress response triggered by ultraviolet B (UVB) (54). In addition, mutations in the SETD1A gene have been reported to be associated with early-onset seizures, which may be related to the fact that the expression of USP39 is reduced by the regulation of the mutant SETD1A gene, which in turn leads to neurodevelopmental disorders (63). Notably, among the many research articles on USP39, the role of USP39 in the development of cancer is particularly notable. Studies have shown that USP39 is abnormally expressed in various types of malignancies and is strongly associated with tumor stage and patient prognosis. A detailed overview of the specific mechanisms of action of USP39 in various cancers is provided below (**Table 2**).

**Table 2:** Substrates and mechanisms associated with USP39 in multiple cancers.

Cancers	Relevant molecule	Involved mechanism	Ref
Hepatocellular carcinoma	FoxM1	USP39 induces G2/M phase block by regulating precursor mRNA splicing of FoxM1.	(72)
	SIRT7	USP39 interacts with SIRT7 and completes deacetylation of USP39, promoting USP39 stability and accelerating HCC cell proliferation and tumorigenesis.	(73)
	ZEB1	USP39 inhibits ZEB1 degradation through its deubiquitylation function to promote HCC progression.	(66)
	β-catenin	USP39 stabilizes β-catenin levels through deubiquitylation to foster advancement of HCC.	(34)
	TRIM26	USP39 regulates TRIM26 precursor mRNA splicing and maturation, reduces TRIM26 ubiquitination and indirectly promotes HCC proliferation and invasion.	(34)
	SP1	USP39 stabilizes SP1 protein and prolongs its half-life by promoting its deubiquitylation pathway to regulate cell cycle and tumor growth in HCC cells.	(68)
	PFKL	USP39 deubiquitinates PFKL to increase the stability of PFKL protein to enhance aberrant glycolysis in HCC cells.	(77)
	SRSF6/HNRNPC	USP39 regulates exon inclusion/exclusion by interacting with SRSF6 or HNRNPC in a position-dependent manner and selectively regulates splice sites to modulate HCC progression.	(78)
Non-small cell lung cancer	miR-381	miR-381 negatively regulates USP39 expression to control tumor proliferation and invasion.	(82)
	MRPL35	USP39 stabilizes MRPL35 expression by deubiquitylation to promote proliferation, invasion and glutamine metabolism in NSCLC cells.	(83)
Breast cancer	CHEK2	USP39 regulates cancer-associated tumor suppressors including CHEK2 and induces tumorigenesis.	(87)
	FOXM1	USP39 and FOXM1 form a negative feedback loop that co-controls breast cancer cell proliferation.	(88)
Ovarian cancer	β-catenin	USP39 promotes EMT through beta-collagen signaling and regulates ovarian cancer.	(90)
	HMGA2	USP39 promotes efficient splicing of the oncogenic transcription factor HMGA2 at 5’ and 3’ splice sites and increases the malignancy of ovarian cancer cells.	(92)
Colorectal cancer	p21	USP39 regulates p21 stability to modulate cancer progression.	(96)
	p53	USP39 modulates cisplatin-induced oxidative stress and DNA damage responses by regulating p53 stability.	(98)
Glioma	TAZ	USP39 regulates TAZ protein expression by inducing TAZ mRNA maturation and exerts USP39 oncogenic properties in glioma.	(100)
	ADAM9	USP39 promotes ADAM9 pre-mRNA maturation, which alters integrin β1 expression and activity and promotes migration and invasion of human glioma cells.	(101)
	Cyclin B1	USP39 stabilizes Cyclin B1 expression by deubiquitinating it and promotes G2/M cell cycle transition and glioma cells proliferation.	(35)
	miR-370-3p	USP39 is involved in the regulation of GBM progression through the circCLSPN-miR-370-3p-USP39 axis.	(104)
Renal cell carcinoma	SRSF1	USP39 promotes SRSF1 phosphorylation and regulates selective splicing of VEGF-A.	(107)
Prostate cancer	EGFR	Knockdown of USP39 inhibits the malignant transformation of PCs by interrupting the transcriptional elongation, maturation and stability of EGFR mRNA.	(111)
Gastric cancer	miR-133a	miR-133a directly targets and negatively regulates USP39.	(114)
Pancreatic cancer	miR-133a	miR-133a directly targets USP39 and promotes pancreatic cancer progression through the AKT signaling pathway.	(116)
Esophageal squamous cell carcinoma	mTORC2	USP39 selectively promotes Rictor mRNA splicing and maturation to regulate mTORC2-mediated signaling and promote ESCC.	(119)
Head and neck squamous cell carcinoma	STAT1	USP39 downregulation inhibits HNSCC survival and migration by suppressing STAT1 expression.	(120)
Nasopharyngeal carcinoma	miR-26b-3p	USP39 plays a role in contributing to NPC through the LINC00520/miR-26b-3p/USP39 pathway.	(122)
Osteosarcoma	miR-1281	miR-1281 targets USP39, which in turn regulates endoplasmic reticulum stress-induced apoptosis.	(129)
Cervical squamous cell carcinoma	SIRT7	USP39 promotes CSCC through a SIRT7/USP39/FOXM1 positive feedback loop.	(130)
Endometrial cancer	PGK1	USP39 activates the PI3K/AKT/HIF-1α signaling pathway, stimulates glycolysis and promotes malignant EC progression.	(131)

## In hepatocellular carcinoma

Primary liver cancer is one of the leading causes of global cancer mortality, accounting for over 700,000 deaths annually. It is the third most commonly occurring cancer and the fourth most common cause of cancer-related mortality worldwide (64). Of these, HCC represents the most prevalent form of liver cancer, accounting for approximately 85% to 90% of all liver malignancies (65). In HCC tissues, USP39 expression is upregulated at both the mRNA and protein levels. Furthermore, its overexpression significantly correlates with tumor stage, histological grade, and size. It also serves as an independent prognostic factor for overall survival in HCC (66–69).

The knockdown of USP39 resulted in the inhibition of proliferation and colony formation in SMMC-7721 cells, leading to cell arrest in the G2/M phase and the induction of apoptosis. This ultimately led to the inhibition of HCC cell growth. Furthermore, USP39 knockdown resulted in the inhibition of tumor growth in xenografts in nude mice, accompanied by a reduction in the expression levels of forkhead box protein M1 (FoxM1) and its target genes polo-like kinase 1, cyclin B1, and centromere protein A (70, 71). FoxM1 is a member of the forkhead transcription factor family and plays a role in the development of the G2/M phase of HCC cells by regulating the expression of target genes in the G2/M phase. These target genes are involved in mitotic entry and the execution of the mitotic program (72). The results indicate that the knockdown of USP39 inhibits the growth of HCC *in vitro* and *in vivo*, potentially by regulating the splicing of precursor mRNA to induce a G2/M phase block. Previously, it was reported that USP39 could be acetylated by histone acetyltransferase MYST1, and the acetylated USP39 was subsequently degraded by E3 ubiquitin ligase VHL-mediated proteasomal degradation. Conversely, USP39 interacted with and deacetylated USP39 via SIRT7, a SIRT-family NAD+-dependent deacetylase, thereby increasing USP39 stability and promoting HCC cell proliferation and tumor formation *in vitro* and vivo (73).

Zinc-finger E-box-binding homeobox 1 (ZEB1) has been identified as a crucial inducer of epithelial-to-mesenchymal transition (EMT), which contributes to promoting tumor proliferation and metastasis (74). The accumulating evidence indicates that ZEB1-mediated EMT makes a significant contribution to the etiology of HCC. It was found that the deubiquitinating enzyme USP39 and the E3 ligase TRIM26, a recently identified tumor suppressor in HCC, together regulate HCC cell proliferation and migration by modulating the ubiquitination level of ZEB1 (66). Specifically, USP39 promotes HCC progression by inhibiting the degradation of ZEB1 through its deubiquitylation function, whereas TRIM26 exerts tumor suppressor functions by ubiquitinating degraded ZEB1. This novel finding provides a new strategy for targeting either USP39 or TRIM26 for the treatment of HCC cases exhibiting aberrant ZEB1 expression levels. It is interesting to note that USP39 and TRIM26 not only co-regulate ZEB1 but also interact directly with each other, as demonstrated by immunoprecipitation and immunofluorescence staining assays. This provides a new perspective for exploring the underlying mechanisms of HCC. In a subsequent study, Wang et al. discovered that USP39 directly interacts with β-catenin and stabilizes β-catenin levels through deubiquitylation, thereby promoting HCC progression (34). β-catenin is a critical molecule in the Wnt/β-catenin signaling pathway, and its aberrant expression or activation has been linked to a multitude of tumors. β-catenin accumulation is closely associated with HCC progression and poor prognosis (75). Furthermore, the knockdown of USP39 increased TRIM26 mRNA and protein levels. Subsequent studies showed that USP39 regulates TRIM26 precursor mRNA splicing and maturation, and reduces TRIM26 protein levels, at least in part, by inhibiting the splicing of its precursor mRNAs. This affects TRIM26-induced ubiquitylation of β-catenin and indirectly promotes HCC proliferation and invasion.

Recent studies have identified specificity protein 1 (SP1) as a novel substrate for USP39, with SP1 demonstrating the capacity to effectively reverse apoptosis and cell cycle arrest induced by USP39 knockdown (68). USP39 regulates the cell cycle and tumor growth of HCC cells by facilitating the deubiquitylation pathway of the SP1 protein, stabilizing its protein level, and prolonging its half-life. Dynein axonemal assembly factor 5 (DNAAF5) is an early scaffold for the assembly of dynamin complexes in motor cilia (76). It was reported that the expression of DNAAF5 was markedly elevated in HCC tissues, which was found to be negatively correlated with the poor prognosis of HCC patients (77). From a mechanistic perspective, DNAAF5 directly binds to PFKL (a key rate-limiting enzyme in glucose metabolism) and recruits USP39, which deubiquitinates PFKL to enhance the stability of the PFKL protein, thereby enhancing aberrant glycolysis and accelerating the malignant progression of HCC in HCC cells. AS serves as a pivotal mediator in the processing of RNA. USP39, a spliceosomal protein that functions in the mid/late stages, effectively regulates splice site selection and has been demonstrated to promote hepatocarcinogenesis in transgenic mice (78). In human HCC cells, USP39 has been shown to facilitate tumor proliferation in a spliceosome-dependent manner, which is partly mediated by the oncogenic splice switch in the focal adhesion protein gene KANK2. Concerning the molecular mechanisms involved, Zheng et al. present a novel regulatory model that is conserved in humans and mice. This model illustrates how USP39 regulates exon inclusion/exclusion by interacting with SRSF6 or HNRNPC in a position-dependent manner. These findings indicate that USP39 may have potential as a tumor biomarker and therapeutic target.

## In non-small cell lung cancer

Lung cancer remains the second most common cancer worldwide and is mainly divided into two types: NSCLC and small cell lung cancer (SCLC). Approximately 85% of patients present with the histological subtype of NSCLC (79). In human lung cancer tissues, the expression of USP39 is typically higher than that observed in normal tissues. Knockdown of USP39 significantly reduces the proliferation and colony-forming ability of lung cancer cell lines 95D and A549, leading to cell cycle arrest in the G2/M phase and inducing apoptosis (80, 81). Deletion of USP39 has been shown to block the activation of Akt, mTOR, p53, and PARP signaling pathways. MiR-381 is a member of the microRNA (miRNA) family, which is up-regulated in NSCLC expression and involved in the regulation of cell proliferation (82). USP39 is a direct target of miR-381. miR-381 controls tumor proliferation and invasion by negatively regulating USP39 expression, providing a new idea for targeted therapy of NSCLC. The p53 signaling pathway and its regulators perform a salient function in the anti-tumor development of lung cancer (81). In particular, the downregulation of USP39 has been shown to activate the p53 pathway, as evidenced by the upregulation of p21, cleaved-cas3, and cleaved-cas9, and the downregulation of CDC2 and CycinB1. This results in cell cycle arrest and apoptosis. Furthermore, knockdown of USP39 inhibited migration and invasion of A549 and HCC827 cells through activation of the p53 pathway and downregulation of MMP2 and MMP9. Interestingly, a recent study showed that the knockdown of the mitochondrial ribosomal protein L35 (MRPL35) inhibits NSCLC progression. Meanwhile, USP39 stabilizes the level of MRPL35 through deubiquitylation, which in turn upregulates the expression of solute carrier family 7 member 5 (SLC7A5) and promotes proliferation, invasion, and glutamine metabolism in NSCLC cells (83).

## In breast cancer

BC is the most predominant malignant tumor in women, with about 2.3 million new cases worldwide in 2020, accounting for 11.7% of all cancer incidences (84). The expression level of the USP39 protein is elevated in human breast cancer tissues. RNAi-mediated USP39 has been proven to substantially diminish the proliferation and colony-forming capacity of McF-7 cells. Additionally, the decreased expression of USP39 causes G0/G1 phase arrest and apoptosis (85). Furthermore, the inhibition of cell growth was observed in triple-negative breast cancer (TNBC) cells by doxycycline-regulated lentiviral vector-induced USP39 downregulation (86). In addition, an exome sequencing study in BC patients demonstrated that USP39 regulates cancer-associated tumor suppressors, including CHEK2, and has a tumor-inducing effect (87). It is worth noting that, as in HCC, USP39 interacts with FOXM1 in BC regulating ubiquitination and stabilizing FOXM1 through competitive bonding with the E3 ubiquitin ligase APC/Cdh1, which regulates BC cell proliferation (88). The expression of USP39 and FOXM1 was found to be upregulated and positively correlated in BC, indicating that USP39 may possess potential prognostic value in this disease. Increased expression of USP39 has been observed to reduce the ubiquitination of FOXM1, enhance the transcriptional activity of FOXM1, and regulate the expression of the downstream genes CDC25B and PLK1. Interestingly, USP39 is also subject to FOXM1 regulation Therefore, it was indicated that USP39 and FOXM1 constitute a negative feedback loop that co-regulates the proliferation of BC cells, thereby offering a novel approach to targeting the USP39-FOXM1 axis for the treatment of BC.

## In ovarian cancer

OC ranks as the second most common gynecological malignancy and remains one of the leading causes of death in women worldwide (89). High expression of USP39 in OC tissues is associated with worsening TNM stage and poor prognosis (90–92). Analyses of the functional effects of USP39 inhibition revealed that this approach significantly suppressed ovarian cancer cell growth and migration, induced cell cycle G2/M phase arrest, and impaired clone formation. Yan et al. demonstrated that USP39 regulates the G2/M phase by interfering with the P53/P21 pathway and promotes EMT through the β-catenin/LEF/TCF/slug pathway, collectively regulating the proliferation and metastasis of OC (90). Carboplatin is a conventional drug used to treat OC. USP39 confers chemoresistance to OC cells through the AKT/extracellular signal-regulated kinase (ERK) signaling pathway. Furthermore, the expression of USP39 promotes carboplatin resistance and the malignant phenotype of OC (91). It may therefore be posited that the development of strategies targeting USP39 may prove an efficacious means of overcoming treatment resistance in OC patients. A recent study found that USP39 acts as a splicing factor that is transcriptionally activated by the oncogene protein c-MYC in OC cells and colocalizes with spliceosomal components in nuclear speckles (92). USP39 gene deletions can cause an overall impairment of splicing function, characterized by exon skipping and inappropriate retention of introns and intergenic regions. Furthermore, USP39 facilitates the splicing of the oncogenic transcription factor HMGA2 (high-mobility group AT-hook 2) at the 5’ and 3’ splice sites, thereby augmenting the malignant potential of OC cells.

## In colorectal cancer

CRC is one of the most prevalent malignant tumors of the gastrointestinal tract worldwide, ranking third among all cancers in terms of morbidity and mortality (93). USP39 expression is upregulated in CRC tissues and cell lines and contributes to tumorigenesis both *in vivo* and ex vivo, affecting patient survival and prognosis (94–96). USP39 knockdown inhibits CRC cell proliferation, colony-forming ability, and cell cycle progression.

Xing et al. found that the silencing of USP39 inhibits the proliferation of CRC cells and induces apoptosis by activating the caspase cascade and upregulating p53 expression (94). It is evident from numerous studies that aberrant activation of the Wnt/β-catenin signaling pathway is instrumental in human tumor formation, particularly that of CRC (97). The knockdown of the USP39 gene was observed to result in a reduction in the expression of four key proteins of the Wnt/β-catenin pathway, including β-catenin, TCF4, MMP2, and MMP9. This, in turn, was found to regulate the progression of CRC (95). In colon cancer, USP39 regulates colon cancer progression in a p21-dependent manner through the P53/P21/CDC2/CyclinB1 axis. Additionally, it regulates p21 stability by modulating the promoter activity of p21 through the RS and USP structural domains of USP39 (96).

The protein encoded by the USP39 gene is overexpressed in a wide range of malignancies and is involved in the regulation of cellular apoptosis, the DNA damage response, and platinum resistance. For example, previous studies have demonstrated that USP39 plays a role in carboplatin resistance in ovarian cancer and in regulating chemoresistance to cisplatin by stabilizing CHK2 in human lung cancer cells (40, 90). Similarly, cisplatin, an effective chemotherapeutic agent for the treatment of colon cancer, has been found to correlate with the sensitivity of colon cancer cells to cisplatin in a manner that is dependent on USP39 (98). It can be postulated that USP39 participates in the regulation of cisplatin-induced oxidative stress and DNA damage response via the modulation of p53 stability, which in turn regulates cisplatin-induced apoptosis in colon cancer cells. Therefore, it may be proposed that USP39 represents a potential molecular target for cisplatin therapy in the treatment of colon cancer.

## In glioma

Gliomas are primary tumors of the central nervous system. Among these, glioblastoma (GBM) is the most common and fatal primary malignant brain tumor in adults (99). In patients with gliomas, high USP39 expression is associated with a poor prognosis. Furthermore, the knockdown of USP39 has been shown to significantly inhibit the migration and invasion of glioma cells *in vitro (* 100, 101).

TAZ, also known as WWTR1 (transcriptional regulator 1 containing the WW structural domain), has been identified to exhibit elevated expression and activity in a range of human cancers (102). The expression of TAZ proteins is regulated by USP39, which induces the maturation of TAZ mRNA and thereby exerts oncogenic properties in gliomas (100). This process is independent of the classical Hippo signaling pathway; rather, it is due to the enhancement of the splicing ability of the TAZ pre-mRNA by USP39. Interestingly, Xiao et al. found that USP39 also directly binds to ADAM9 mRNA and promotes its pre-mRNA maturation, thereby altering the expression and activity of integrin β1 and promoting migration and invasion of human glioma cells (101). ADAM9, as a member of the disintegrin and metalloproteinase family, is involved in a wide range of solid tumor cell migration intercellular interactions and other biological processes (103). Furthermore, it was discovered that USP39 functions not only as a splicing factor and regulator of mRNA maturation but also as a novel deubiquitinating enzyme of Cyclin B1, thereby participating in the proliferation of tumor cells (35). Cyclin B1 is overexpressed in gliomas, which correlates positively with pathological grading. The deubiquitinating enzyme USP39 acts directly on Cyclin B1, promoting its stabilization and expression, the transition from the G2 to M phase of the cell cycle, the proliferation of glioma cells, and tumor growth *in vivo*.

In addition, USP39 was identified as a regulator of GBM progression through the circCLSPN-miR-370-3p-USP39 axis. circCLSPN (a cyclic RNA derived from the CLSPN gene) acts as an oncogene in human GBM, whereas miR-370-3p exerts a tumor-suppressive function. When the expression of circCLSPN and USP39 is down-regulated, miR-370-3p is then able to target USP39, thus partially reversing the inhibitory effect of miR-370-3p overexpression on GBM cell growth, migration, and invasion (104).

## In renal cell carcinoma

RCC accounts for approximately 3% of adult cancers, with clear cell RCC representing the predominant cancer type, comprising approximately 70-80% of all RCC cases (105). Elevated levels of USP39 mRNA expression in RCC are negatively correlated with the survival of RCC patients, and USP39 expression is identified as an independent risk factor affecting the survival of RCC patients (106). The silencing of USP39 was observed to significantly inhibit the proliferation and invasion of RCC cells, while also inducing cell cycle arrest and apoptosis (107). Furthermore, the downregulation of USP39 resulted in the inhibition of the Akt/ERK signaling pathway in RCC cells. Pan et al. identified that the ZnF-UCH1-UCH2 complex structural domain of USP39 directly mediated its binding to SRPK1 (serine/arginine-rich protein-specific kinase 1) and SRSF1 (serine/arginine-rich splicing factor 1), facilitating SRSF1 phosphorylation and regulating the alternative splicing of VEGF-A (vascular endothelial growth factor) (107). The inhibition of the AS of VEGF-A165b (an anti-angiogenic factor) by USP39 is responsible for the promotion of malignant proliferation and angiogenesis in RCC.

## In prostate cancer

PC is a commonly occurring malignancy in men, with an estimated 10 million men diagnosed with PC each year worldwide, representing a rate of 1.3 million new cases per year (108). Small ubiquitin-like modifier (SUMO) is a protein modification pathway that regulates a variety of biological processes, including cell division, DNA replication/repair, signaling, and cellular metabolism (109). It was identified that USP39 is a novel SUMOylated protein, with K6, K16, K29, K51, and K73 representing the SUMOylation sites of USP39. The proliferative effects of USP39 on androgen-dependent and independent PC cells are promoted through mutations in these SUMO sites (110). Furthermore, Huang et al. discovered that USP39 was overexpressed in PC tissues, exhibited a positive correlation with the Gleason score, and served as an independent risk factor for biochemical recurrence (111). The knockdown of USP39 has been proven to inhibit the malignant transformation of PC by interrupting the transcriptional elongation, maturation, and stability of EGFR mRNA.

## In other cancers

Gastric cancer (GC), one of the most prevalent types of malignancy, shows a trend toward younger incidence, and most patients are diagnosed at an advanced stage (112). In GC tissues and cells, elevated levels of USP39 expression are associated with poor prognosis (113, 114). Knockdown of USP39 significantly reduces the proliferation and colony-forming ability of MGC80–3 cells and inhibits the growth of GC cells by regulating the cleavage of polyadenosine diphosphate ribose polymerase (PARP) inducing G2/M-phase block (113). Furthermore, USP39 was identified as a direct target of miR-133a, and an inverse relationship between the two was observed. The upregulation of miR-133a expression and/or the downregulation of USP39 inhibited the proliferation of gastric cancer cells (114). Interestingly, in pancreatic cancer, miR-133a has also been reported to directly target USP39, thereby promoting pancreatic cancer progression through the activation of the AKT signaling pathway (115, 116). Esophageal squamous cell carcinoma (ESCC) is the sixth most common cause of cancer-related mortality worldwide, with more than half of all cases occurring in China (117). USP39 in ESCC not only drives the growth of cancer cells but also correlates with chemotherapy resistance. As a splicing factor, USP39 interacts with multiple spliceosome components (e.g., EFTUD2, PRPF3, SART1, DDX23, and hnRNPU) to regulate AS events (118). Zhao et al. found that USP39 regulates the splicing and maturation of Rictor mRNAs, a component of mTORC2 (a type of the mTOR complex), through selectively promoting the mTORC2-mediated signaling, which in turn promotes ESCC (119).

**Figure 3:**
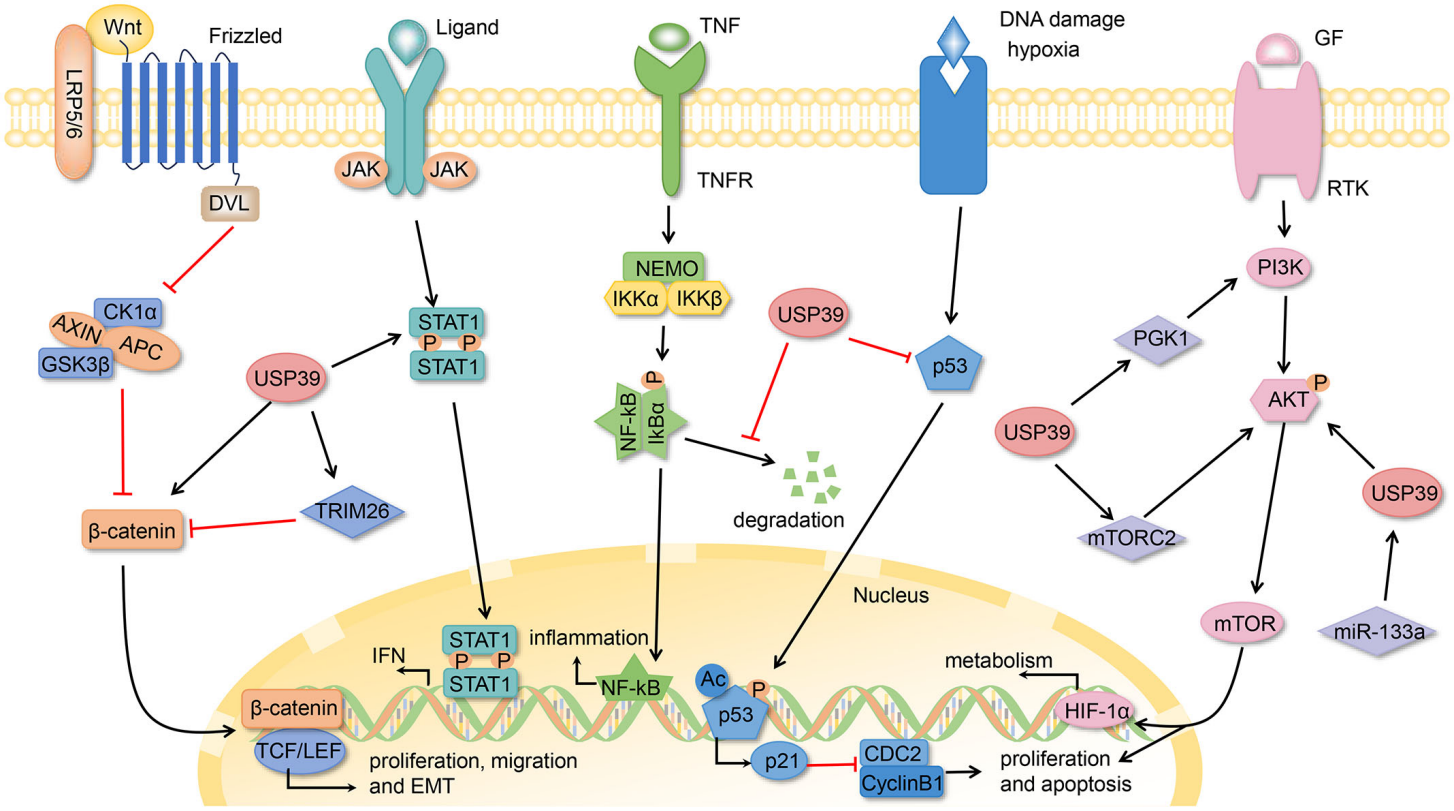
The USP39-related signaling pathways.

USP39 is frequently overexpressed in a range of cancers, including head and neck squamous cell carcinoma (HNSCC), oral squamous cell carcinoma (OSCC), nasopharyngeal carcinoma (NPC), medullary thyroid carcinoma (MTC), melanoma, and leukemia. It has been reported to significantly promote cancer cell proliferation and invasion *in vitro* and vivo (120–125). In HNSCC, USP39 inhibits HNSCC survival and migration by suppressing the expression of STAT1 (signal transducer and activator of transcription) (120). NPC is highly prevalent in East and Southeast Asia (126). USP39 exerts its oncogenic effects on NPC through the LINC00520/miR-26b-3p/USP39 pathway. LINC00520 (long non-coding RNA 520) functions as a competitive endogenous RNA for miR-26b-3p, thereby activating the USP39 signaling pathway to exert an oncogenic effect (122). In melanoma, USP39 is involved in melanoma progression by regulating cell cycle and apoptosis through the ERK1/2 signaling pathway (124). Osteosarcoma (OS) is the most prevalent primary bone cancer in childhood and adolescence, originating from primitive bone-forming mesenchymal cells (127). Additionally, USP39 has been identified as a risk factor for OS. Down-regulation of USP39 expression results in reduced cell proliferation and clone formation, while promoting apoptosis through PARP cleavage (128). Furthermore, Jiang et al. provided additional evidence that USP39 participates in osteosarcoma development by regulating apoptosis during miR-1281-induced endoplasmic reticulum stress (129). In cervical squamous cell carcinoma (CSCC), the deacetylation of USP39 by SIRT7 leads to the promotion of SIRT7 expression via FOXM1-mediate transcription, which subsequently enhances the development of CSCC (130). Moreover, the SIRT7/USP39/FOXM1 positive feedback loop has been shown to promote autophagy and inhibit oxidative stress in CSCC. In endometrial cancer (EC), lactate production promotes histone lactosylation, which in turn stimulates USP39 expression (131). USP39 promotes the malignant progression of EC by interacting with and stabilizing PGK1 (phosphoglycerate kinase 1), activating the PI3K/AKT/HIF-1α signaling pathway, and stimulating glycolysis (**Figure 3**).

## Conclusion and future perspectives

Ubiquitination and deubiquitylation are two basic forms of post-translational modification of proteins, which play crucial roles in numerous physiological and pathological processes. As a member of USPs, USP39 has been widely studied and focused on its structure and biological functions in the past decade. USP39 mainly contains two structural domains, C_2_H_2_ ZnF and UCH, which are responsible for recognizing and counteracting the ubiquitination of specific substrates. The multifunctionality of USP39 is closely related to its unique structural domains, which are involved in a variety of activities, including DNA damage repair, RNA splicing, viral immune response, inflammatory response, and vascular remodeling, and plays an important role in the development of many diseases. In addition, USP39 acts as an activator or inhibitor in disease states by regulating multiple signaling pathways such as PI3K/Akt, Wnt/β-catenin, P53/P21, and NF-κB, suggesting that USP39 may be a key regulator affecting multiple biological functions.

Interestingly, we unexpectedly found that although USP39 belongs to deubiquitinating enzymes, its deubiquitinating enzyme structural domain did not exhibit the activity of typical DUBs, which is in apparent contradiction to the experimentally observed deubiquitinating enzyme activity of USP39. This phenomenon triggered a rethinking of the function of the USP39 structural domains: are there unknown structural domains other than C_2_H_2_ ZnF and UCH, or might there be some kind of synergistic interaction between these known structural domains to confer USP39 deubiquitinating enzyme activity? This query is currently inconclusive and future studies are urgently needed to reveal the exact mechanism behind it.

Of particular interest is the critical role of USP39 as a splicing factor in organisms, which has been extensively highlighted in numerous research studies. Specifically, USP39 regulates the rearrangement of immunoglobulin genes through a spliceosome-dependent mechanism, thereby affecting B cell maturation and function; it also maintains hepatic autophagy and lipid homeostasis through AS processes. In addition, USP39 takes a role in regulating the splicing and maturation of protein precursor mRNAs, a function that is particularly prominent in promoting the malignant progression of cancers such as HCC and OC. These findings highlight the functional differences between USP39 and conventional DUBs, revealing the broad and far-reaching impact of USP39 as a splicing factor in the regulation of gene expression, the maintenance of cellular function, and disease genesis and therapy. Therefore, using these studies as a starting point, future explorations may open up new paths and guide the direction of in-depth research on USP39.

With the increasing research on the relationship between USP39 and cancer, a large number of studies have shown that USP39 is overexpressed in a variety of cancers such as CRC, NSCLC, and HCC, and that it can be a valuable biomarker for diagnostic and prognostic purposes. Although the regulatory mechanisms of USP39 in tumors are complex, like most members of the USP family, it can act as a tumor promoter to promote cancer, both through cell-intrinsic pathways and by fostering an immunosuppressive microenvironment, for instance via splicing-mediated regulation of immune checkpoints like CTLA-4. This singular oncogenic characteristic, particularly its role in immune evasion, distinguishes it and makes it a compelling therapeutic target. However, to date, no robust evidence supports anti-cancer functions for USP39, and this singular oncogenic characteristic distinguishes it from the general oncogenic properties of USPs. For example, USP9 X, USP10, USP18, USP22, and USP28 may play diametrically opposite roles in different cancers (132). Consequently, more studies are necessary to fully understand the role of USP39 in cancer and to explore whether USP39 has only pro-cancer effects or its potential anti-cancer effects have not yet been identified. If USP39 is only pro-cancer, it would provide a strong scientific basis for the development of its inhibitors and raise expectations.

The ubiquitin-proteasome system (UPS) serves as the primary pathway for selective protein degradation in eukaryotic cells (133). Target proteins are tagged with polyubiquitin chains through an E1-E2-E3 enzyme cascade, then recognized and degraded by the 26S proteasome. The 19S regulatory particle of the proteasome contains deubiquitinating enzymes (DUBs) responsible for substrate recognition, partial deubiquitylation, and unfolding, while the 20S core particle carries out hydrolysis. The UPS maintains proteostasis and regulates critical cellular processes such as the cell cycle and apoptosis. Dysregulation of the UPS is associated with various cancers and chemotherapy resistance (e.g., 5-fluorouracil resistance). As key regulators of the UPS, inhibitors targeting DUBs are currently being evaluated in clinical trials as potential anticancer agents. In particular, DUBs of the USPs family have shown great potential in cancer therapy, such as P5091 (USP7 inhibitor) (134), Spautin-1 (USP10 inhibitor) (135), AZ1 (USP28 inhibitor) (136), and EOAI3402143 (USP5/USP9X dual inhibitor) (137). However, as of today, the development of inhibitors targeting USP39 remains insufficient, and, unfortunately, no small molecule USP39 inhibitors are currently available for clinical use. The absence of catalytic activity in USP39’s UCH domain complicates traditional active-site inhibitor design. Alternative strategies targeting its ZnF-UBP domain or protein-protein interfaces (e.g., with SRSF6/HNRNPC) warrant exploration. In addition, the problems of insufficient specificity, inefficient inhibition, and potential normal cytotoxicity faced by small-molecule targeted therapies have limited the development of drugs against USP. Therefore, the development of highly specific inhibitors based on the binding region of USP39 to downstream molecules, or the consideration of combining checkpoint inhibitors to provide new strategies for cancer treatment, is an important direction for future research.

In summary, studies of USP39 have not only elucidated its unique structural features and biological functions, but also revealed the key role of the enzyme in cancer and other diseases, laying the foundation for the development of novel therapeutic strategies. Looking forward, continued and in-depth studies on the function of the structural domains of USP39, its splicing regulatory mechanism, and its specific role in cancer development are of great significance for the design of more effective therapeutic means.

## Glossary

AS: alternative splicing
BC: breast cancer
C_2_H_2_ ZnF: zinc finger ubiquitin-binding structural domain
CHK2: checkpoint kinase 2
CPC: Chromosomal Passenger Complex
CRC: colorectal cancer
CSCC: cervical squamous cell carcinoma
DDR: DNA damage response
DNAAF5: dynein axonemal assembly factor 5
DUBs: deubiquitinating enzymes
EMT: epithelial-to-mesenchymal transition
ERK: extracellular signal-regulated kinase
ESCC: esophageal squamous cell carcinoma
FoxM1: forkhead box protein M1
GBM: glioblastoma
GC: gastric cancer
GRHL: granular head-like factor
HCC: hepatocellular carcinoma
HMGA2: high-mobility group AT-hook 2
HNSCC: head and neck squamous cell carcinoma
Hsf1: heat shock transcription factor 1
IFN: interferon
ISGs: interferon-stimulated genes
ISRE: interferon-stimulated response element
JAMMs: JAB1/MPN+/MOV34 motif proteases
LINC00520: long non-coding RNA 520
MCPIPs: monocyte chemotactic protein-induced proteins
MINDYs: ubiquitin-containing DUB family-interacting motifs
miRNA: microRNA
MJDs: Machado-Joseph disease protein domain proteases
mRNAs: messenger RNAs
MRPL35: mitochondrial ribosomal protein L35
MTC: medullary thyroid carcinoma
NHEJ: non-homologous end-joining
NPC: nasopharyngeal carcinoma
NSCLC: non-small cell lung cancer
OS: osteosarcoma
OSCC: oral squamous cell carcinoma
OTUs: ovarian tumor proteases
PAR: poly ADP-ribose
PARP: polyadenosine diphosphate ribose polymerase
PARP1: PAR polymerase 1
PC: prostate cancer
PCP: planar cell polarity
PGK1: phosphoglycerate kinase 1
pre-mRNAs: precursor messenger RNAs
PTM: post-translational modification
RCC: renal cell carcinoma
SCLC: small cell lung cancer
shRNA: short hairpin RNA
SLC7A5: solute carrier family 7 member 5
SP1: specificity protein 1
SRPK1: serine/arginine-rich protein-specific kinase 1
SRSF1: serine/arginine-rich splicing factor 1
SUMO: small ubiquitin-like modifier
TNBC: triple-negative breast cancer
Tregs: regulatory T cells
Ub: ubiquitin
UBL: ubiquitin-like
UCH: ubiquitin C-terminal hydrolase
UIM: ubiquitin interaction motif
UPS: ubiquitin-proteasome system
USP39: Ubiquitin Specific Protease-39
USPs: ubiquitin-specific proteases
UVB: ultraviolet B
VSMCs: vascular smooth muscle cells
ZEB1: Zinc-finger E-box-binding homeobox 1
ZnF-UBP: zinc finger ubiquitin-binding protein
ZUFSPs: zinc fingers of proteins with UFM1-specific peptidase structural domains
